# Revolutionary Impact of Nanodrug Delivery on Neuroscience

**DOI:** 10.2174/157015912804143513

**Published:** 2012-12

**Authors:** Reza Khanbabaie, Mohsen Jahanshahi

**Affiliations:** 1 Nanotechnology Research Institute, Babol University of Technology, Babol, Iran; 2 Faculty of Basic Science, Department of Physics, Babol University of Technology, Babol, Iran; 3 Department of Physics, University of Ottawa, Ottawa, Canada; 4 Faculty of Chemical Engineering, Babol University of Technology, Babol, Iran

**Keywords:** Nanodrug, Nanofabrication and purification, Neuroscience, Nervous system, Nano-nervous drugs.

## Abstract

Brain research is the most expanding interdisciplinary research that is using the state of the art techniques to overcome limitations in order to conduct more accurate and effective experiments. Drug delivery to the target site in the central nervous system (CNS) is one of the most difficult steps in neuroscience researches and therapies. Taking advantage of the nanoscale structure of neural cells (both neurons and glia); nanodrug delivery (second generation of biotechnological products) has a potential revolutionary impact into the basic understanding, visualization and therapeutic applications of neuroscience. Current review article firstly provides an overview of preparation and characterization, purification and separation, loading and delivering of nanodrugs. Different types of nanoparticle bioproducts and a number of methods for their fabrication and delivery systems including (carbon) nanotubes are explained. In the second part, neuroscience and nervous system drugs are deeply investigated. Different mechanisms in which nanoparticles enhance the uptake and clearance of molecules form cerebrospinal fluid (CSF) are discussed. The focus is on nanodrugs that are being used or have potential to improve neural researches, diagnosis and therapy of neurodegenerative disorders.

## 1 INTRODUCTION

The delivery of drugs to the nervous system is mainly limited by the presence of two anatomical and biochemical dynamic barriers: the blood–brain barrier (BBB) and blood–cerebrospinal fluid barrier (BCSFB) separating the blood from the cerebral parenchyma [1]. These barriers tightly seal the central nervous system (CNS) from the changeable milieu of blood. With the advancement of electron microscopy it is found that the ultrastructural localization of the blood–brain barrier is correlated with the capillary endothelial cells within the brain [2]. The BBB inhibits the free paracellular diffusion of water-soluble molecules by an elaborate network of complex tight junctions (TJs) that interconnects the endothelial cells. Similar to the endothelial barrier, the morphological correlate of the BCSFB is found at the level of unique apical tight junctions between the choroid plexus epithelial cells inhibiting paracellular diffusion of water-soluble molecules across this barrier [1, 3]. Beside its barrier function, it allows the directed transport of ions and nutrients into the cerebrospinal fluid (CSF) and removal of toxic agents out of the CSF using numerous transport systems.

One of the most challenging steps in neuroscience researches and therapy is the availability of techniques to penetrate these permeability barriers and delivering drugs to the CNS. Several strategies have been used to circumvent the barriers inhibiting CNS penetration. These strategies generally fall into one or more of the following three categories: manipulating drugs, disrupting the BBB (BBBD) and finding alternative routes for drug delivery. Drug manipulation methods include: Lipophilic Analogs, prodrugs, chemical drug delivery systems (CDDS), Carrier-mediated transport (CMT) and Receptor-mediated drug delivery. The drug manipulating strategy has been frequently employed, but the results have often been disappointing [4-6]. All of these methods have major limitations: they are invasive procedures, have toxic side effects and low efficiency, and are not sufficiently safe [7]. Two methods for disrupting the BBB have been reported: osmotic blood-brain barrier disruption and biochemical blood-brain barrier disruption. However, these procedures also break down the self-defense mechanism of the brain and make it vulnerable to damage or infection from all circulating chemicals or toxins. Since the above techniques aim to enhance the penetration of drugs to the CNS *via *circulatory system, they will increase the penetration of drugs throughout the entire body. This will frequently result in unwanted systemic side effects. In the other hand, systemically administered agents must penetrate the BBB to enter the CNS, which is a difficult task. Despite advances in rational CNS drug design and BBBD, many potentially efficacious drug molecules still cannot penetrate into the brain parenchyma at therapeutic concentrations. The alternative strategy to enhance CNS penetration of drug molecules is based on methodology that does not rely on the cardiovascular system. These strategies circumvent the BBB altogether and do not need drug manipulation to enhance BBB permeability and/or BBBD. Using alternative routes to deliver drugs to the CNS, e.g. intraventricular/intrathecal route and olfactory pathway, is one of these strategies.

One strategy for bypassing the BBB that has been studied extensively both in laboratory and in clinical trials is the intralumbar injection or intreventricular infusion of drugs directly into the CSF. Compared to vascular drug delivery, intra-CSF drug administration theoretically has several advantages. Intra-CSF administration bypasses the BCB and results in immediate high CSF drug concentrations. Due to the fact that the drug is somewhat contained within the CNS, a smaller dose can be used, potentially minimizing systemic toxicity. Furthermore, drugs in the CSF encounter minimize protein binding and decrease enzymatic activity relative to drugs in plasma, leading to longer drug half-life in the CSF. Finally, since the CSF freely exchanges molecules with the extracellular fluid of the brain parenchyma, delivering drugs into the CSF could theoretically result in therapeutic CNS drug concentrations [7, 8]. However, for several reasons this delivery was not as successful as predicted. These include a slow rate of drug distribution within the CSF and increase in intracranial pressure associated with fluid injection or infusion into small ventricular volumes. 

Another CNS drug delivery route is the intranasal route. In this method drugs are transported intranasally along olfactory sensory neurons to yield significant concentrations in the CSF and olfactory bulb. An obvious advantage of this method is that it is noninvasive relative to other strategies. This method has received relatively little attention, since there are difficulties that have to be overcome. Among these obstacles is an enzymatically active, low pH nasal epithelium, the possibility of mucosal irritation or the possibility of large variability caused by nasal pathology, such as common cold. 

Based on the advantages and disadvantages of aforementioned strategies, researchers are still looking for novel and better methods of CNS drug deliveries. The most direct way of circumventing the BBB is to deliver drugs directly to the brain interstitium which mainly includes the use of small colloidal particles like liposomes and nanoparticles [8]. By directing agents uniquely to an intracranial target, interstitial drug delivery can theoretically yield high CNS drug concentrations with minimal systemic exposure and toxicity. Furthermore, with this strategy, intracranial drug concentrations can be sustained, which is crucial in treatment with many chemotherapeutic agents. The basic reason of common acceptance of these carriers is due to their controlled profile or drug release nature as well as due to their selected targeting mechanism. Targeting action maybe due to the steric hindrance created by nano-vectors for achieving targeting ability. These carriers are usually administered through parenteral route and due to their steric phenomenon they conceal themselves from opsonisation event induced by tissue macrophages. By this way they achieve targeting ability to brain and other reticuloendothelial system (RES) organs like liver, spleen, etc. 

Several approaches have been developed for delivering drugs directly to the brain interstitium like injections, catheters, and pumps. One such methodology is the Ommaya reservoir or implantable pump which achieves truly continuous drug delivery. Though interstitial drug delivery to the CNS has had only modest clinical impact, its therapeutic potential may soon be realized using new advances in polymer technologies to modify the aforementioned techniques. Polymeric or lipidbased devices that can deliver drug molecules at defined rates for specific periods of time are now making a tremendous impact in clinical medicine.

Among the strategies of direct drug delivery to the CNS, nanoparticles have attracted considerable interest from the last few decades. It has been shown that nano delivery systems have great potential to facilitate the movement of drugs across barriers (e.g., BBB). Nanosystems employed for the development of nano drug delivery systems in the treatment of CNS disorders include polymeric nanoparticles, nanospheres, nanosuspensions, nanoemulsions, nanogels, nano-micelles and nano-liposomes, carbon nanotubes, nanofibers and nanorobots, solid lipid nanoparticles (SLN), nanostructured lipid carriers (NLC) and lipid drug conjugates (LDC). Although the exact mechanism of barrier opening by nanoparticles is not known, the novel properties such as tiny size, tailored surface, better solubility, and multi-functionality of nanoparticles present the capability to interact with composite cellular functions in new ways. In fact, nanotechnology has now emerged as an area of research for invention of newer approaches for the CNS drug delivery and a revolutionary method to improve diagnosis and therapy of neurodegenerative disorders. 

In this line, an overview of preparation and characterization, purification and separation, loading and delivering of nanodrugs is the first subject of this review. Different types of nanoparticle bioproducts including carbon nanotubes as a drug delivery system and also as a novel tool in neuroscience research are explored. For instance, nanodrug delivery systems like human serum albumin (HSA) nanoparticles, bovine serum albumin (BSA)-Gum Arabic (Acacia) nanoparticles and α-lactalbumin nanoparticles are explained.

The impact of nanotechnology on neuroscience and drug delivery to the central nervous system (CNS) is the subject of the second part of this review. Different mechanisms in which nanoparticles enhance the uptake of molecules both hydrophilic and hydrophobic across the BBB and the impact of various physiochemical parameters of nanoparticles on its uptake and clearance form CSF are discussed. Also nanodrugs that are being used or have potential to improve neural researches, diagnosis and therapy of neurodegenerative disorders are investigated.

## 2 FROM NANOTECHNOLOGY TO NEUROPHARMACOLOGY

Nanotechnology started by the suggestion of a famous physicist, Richard Feynman, that it should be possible, in principle, to make nanoscale machines that "arrange the atoms the way we want", and do chemical synthesis by mechanical manipulation [9, 10]. Nanotechnologies exploit materials and devices with a functional organization that has been engineered at the nanometer scale. In a broad sense, they can be defined as the science and engineering involved in the design, syntheses, characterization, and application of materials and devices whose smallest functional organization in at least one dimension is on the nanometer scale, ranging from a few to several hundred nanometers. A nanometer is roughly the size of a molecule itself (e.g., a DNA molecule is about 2.5 nm long while a sodium atom is about 0.2 nm) [10]. Nanotechnology is not in itself a single emerging scientific discipline but rather a meeting of traditional sciences such as chemistry, physics, materials science, and biology to bring together the required collective expertise needed to develop these novel technologies.

The application of nanotechnology in cell biology and physiology enables targeted interactions at a fundamental molecular level. Nanotechnology, in the context of nanomedicine, can be defined as the technologies for making nanocarriers of therapeutics and imaging agents, nanoelectronic biosensors, nanodevices, and microdevices with nanostructures. It also covers possible future applications of molecular nanotechnology (MNT) and nanovaccinology. Unlike the definition in core nanotechnology field, which restricts the “nano” to at least 1–100 nm in one dimension, nanocarriers in the biomedical field are often referred to as particles with a dimension a few nanometers to 1000 nm [8, 11]. Although, the initial properties of nanomaterials studied were for its physical, mechanical, electrical, magnetic, chemical and biological applications, recently, attention has been geared towards its pharmaceutical application, especially in the area of drug delivery [8]. There are a few challenges in use of large size materials in drug deliveries. Some of these challenges are poor bioavailability, *in vivo* stability, solubility, intestinal absorption, sustained and targeted delivery to site of action, therapeutic effectiveness, generalized side effects, and plasma fluctuations of drugs (see Table **1**). 

The most important innovations are taking place in nanopharmocology and drug delivery which involves developing nanoscale particles or molecules to improve bioavailability. These pharmacological applications of nanotechnology include: the formation of novel nanoscopic entities [11, 27], exploring and matching specific compounds to particular patients for maximum effectiveness; and advanced pharmaceutical delivery systems and discovery of new pharmacological molecular entities; selection of pharmaceuticals for specific individuals to maximize effectiveness and minimize side effects, and delivery of pharmaceuticals to targeted locations or tissues within the body. Examples of nanomaterials include nanotubes and nanofibers, liposomes, nanoparticles, polymeric micelles, block ionomer complexes, nanogels, and dendrimers.

Nanotubes [28, 29] and nanofibers mimic tubular structures that appear in nature, such as rod shaped bacteria or viruses, microtubules, ion channels, as well as axons and dendrites. They are low-dimensional nanostructures, having a very large axial ratio. Properties of a molecule in a nanotube or nanofiber structure can be different from those in the bulk or in other nanomaterials, such as spherical nanoparticles. These materials have a large surface–volume ratio, which results in a high exposure of the material components to the surrounding environment [30]. This makes nanotubes and nanofibers promising structures for biosensing and molecular recognition [31]. However, it provides a way to control drug release through the nanotubes wall, while the large hollow area inside nanotubes provides an excellent storage for drugs and other agents [32]. Furthermore, nanotubes can be synthesized to be open-ended, which can be exploited for certain biological applications. 

Carbon nanotubes (CNTs) was discovered by Iijima [33] which are composed of carbon atoms arranged in hexagonal ring structures similar to graphite, with some five-membered or seven-membered rings providing the structure curvature [29, 34, 35]. CNTs are compatible with biological tissues for scaffolding purposes and the charge carried by the nanotubes can be manipulated to control neurite outgrowth [36, 37]. It has also been suggested that CNTs functionalized with growth factors, such as nerve growth factor or brain-derived neurotrophic factor, can stimulate growth of neurons on the nanotube scaffold [38-40]. In such application the toxicity of CNTs remains an issue that must be overcome [41, 42]. It has been reported that conductive polymer coatings for living neural cells has been generated using poly (3,4-ethylenedioxythiophene) PEDOT nanotubes [43]. The electric conductivity of PEDOT was used to enhance the electrical activity of the tissue with a long range aim of treating CNS disorders, which show sensory and motor impairments. These observations suggested that nanotube and nanofiber scaffolds have potential for neuroregeneration as well as treatment of CNS trauma [27, 44]. Nanomaterials suggest a promising strategy for neuroprotection [45]. Neuroprotection is an effect that may result in salvage, recovery, or regeneration of the nervous system. 

The role of nanotechnology in targeted drug delivery and imaging was discussed in many reviews and papers [46, 47]. As a step towards a realistic system, a brief overview of preparation, characterization, delivery, loading, purification and separation of nanoparticles and nanodrugs are presented herein. In next two sections the fabrication methods of nanoparticle bioproducts and also the delivery systems of nanodrugs are explained. Subsequently we go back to the CNS nanodrugs for research and therapy and the delivery systems of nanodrugs for nervous system.

### 2.1 Preparation of Nanoparticle Bioproducts

Nanoparticle bioproducts can be prepared from a variety of macromolecules such as proteins (albumin, gelatin, legumin, vicillin), polysaccharides (alginate or agarose) and synthetic polymers. These substances have extensive usage in preparation of biomaterial because of their natural properties such as biodegradability and biocompatibility. Among the above mentioned macromolecules, albumin and gelatin have been used widely [48]. There are four main methods for preparation of such nanoparticles:

#### 2.1.1 W/O Emulsification Method

In this process, an aqueous solution from albumin is turned into an emulsion at room temperature and in plant oil (cotton seed oil). Then by a mechanical homogenizer with high speed, a homogeneous emulsion is obtained. There would be a high dispersion for particles through this method. The above emulsion will be added to a high volume of pre-heated oil (over 120°C) drop by drop. This process will result a rapid evaporation of existed water and albumin irreversible destruction and also cause formation of nanoparticles. Fig. (**1**) illustrates the above method. The resulted suspension was put into cold- ice bath. Generally speaking, main methods of preparation nanoparticles from natural macromolecules include W/O emulsification, phase separation in an aqueous medium by addition of a desolvating agent and/or by modification of the pH [48].

#### 2.1.2 Coacervation (Desolvation) Method

The Emulsification method has a disadvantage which is the need for applying organic solvents, for the removal of both the oily residues of the preparation process and the surfactants required for emulsion stabilization. An alternative method called coacervation method in which particles in aqueous will formed by coacervation process and later on will be stabilized by cross linking agent such as glutaraldehyde. Fig. (**2**) shows preparation of albumin nanoparticles by using desolvating agent. 

Coacervation methods have been continuously maintained as one of the most adopted techniques for nanoparticle bioproducts preparation up to now. To ensure this operation a success, careful selection of operation conditions, initial materials etc. remain one of the essential requirements. Researchers from both academy and industry have paid much effort to design better coacervation methods for improved their performances. This method will be deeply discussed once again in section 4.

#### 2.1.3 Thermal Gelation

Thermal gelation is a sequential process that involves heat-induced unfolding followed by protein–protein interactions including hydrogen bonding, electrostatic, hydrophobic interactions and disulfide–sulfydryl interchange reaction. In a study performed by Yu *et al*., spherical core–shell structure nanogels (about 100 nm) were manufactured using thermal gelation method where ovalbumin and lysozyme solutions were mixed at pH 5.3, the pH of the mixture solution was adjusted to 10.3 and the solution was subsequently stirred and heated [49, 50].

#### 2.1.4 Nano Spray Drying

Recently pharmaceutical industries use spray drying for producing a dry powder from a liquid phase. Unlike conventional spray dryers that use rotary atomizers and pressure nozzles for forming the spray droplets, the new nano spray dryer (NSD) utilizes a vibrating mesh technology for fine droplets generation [51]. Basically, the piezoelectric crystal driven spray head is incorporated with a small spray cap that contains a thin perforated membrane (spray mesh) having an array of precise micron-sized holes. When the piezoelectric actuator is driven at an ultrasonic frequency (i.e. 60 kHz), the mesh will vibrate upwards and downwards, injecting millions of precisely sized droplets from the holes and generating the aerosols [50]. The selection of matrix materials depends on many factors including [52]: (a) size of nanoparticles required; (b) inherent properties of the drug, e.g., aqueous solubility and stability; (c) surface characteristics such as charge and permeability; (d) degree of biodegradability, biocompatibility and toxicity; (e) Drug release profile desired; and (f) antigenicity of the final product. 

### 2.2 Characterization of Nanoparticle Bioproducts

Nanoparticle bioproducts are characterized by their mean particle diameter and size distribution, morphology and their surface charge in general [53]. 

#### 2.2.1 Particle Size

It has been shown that particle size and size distribution are the most important characteristics of nanoparticle systems [54]. Many studies have demonstrated that nanoparticles of sub-micron size have a number of advantages over microparticles as a drug delivery system [55]. Generally nanoparticles have relatively higher intracellular uptake compared to microparticles and available to a wider range of biological targets due to their small size and relative mobility. For example, body distribution studies have shown that nanoparticles larger than 230 nm accumulate in the spleen due to the capillary size in this organ [55]. Different *in vitro *studies indicate that the particle size also influences the cellular uptake of nanoparticles [56, 57]. It was also reported that nanoparticles can cross the blood-brain barrier following the opening of tight junctions by hyper osmotic mannitol, which may supply sustained delivery of therapeutic agents for difficult-totreat diseases like brain tumors. In some cell lines, only submicron nanoparticles can be taken up efficiently but not the larger size microparticles. 

Drug release is affected by particle size. Smaller particles have larger surface area, therefore, most of the drug associated would be at or near the particle surface, leading to fast drug release. While, larger particles have large cores which allow more drug to be encapsulated and slowly diffuse out [58]. Smaller particles also have greater risk of aggregation of particles during storage and transportation of nanoparticle dispersion. It is always a challenge to formulate nanoparticles with the smallest size possible but maximum stability. Polymer degradation can also be affected by the particle size. For instance, the rate of PLGA polymer degradation was found to increase with increasing particle size *in vitro *[59].

#### 2.2.2 Particle Morphology

Manipulation of the physicochemical properties of materials at the nanoscale has the potential to revolutionize electronic, diagnostic, and therapeutic applications. Because of the potential large-scale use of nanomaterials, it is important to determine if there is any unique toxicity of the nanoscale materials as compared to the bulk. It is essential for the purposes of interpreting results from cell culture and animal models that the nanomaterials are thoroughly characterized and that correlations are made between observed toxicological responses and the physicochemical characteristics of the materials. The morphology of nanoparticles are examined by two techniques. Atomic force microscopy (AFM) and scanning electron microscopy (SEM) [60, 61]. The atomic force microscope (AFM) or scanning force microscope (SFM) is a very high-resolution type of scanning probe microscope, with demonstrated resolution of fractions of a nanometer, more than 1000 times better than the optical diffraction limit. SEM has the required nanometer resolution for sizing in the submicron range and is invaluable to determine the particle morphology. The electrons interact with the atoms that make up the sample producing signals that contain information about the sample's surface topography, composition and other properties such as electrical conductivity.

#### 2.2.3 Surface Charge

When nanoparticles are administered intravenously, they are easily recognized by the body immune systems, and are then cleared by phagocytes from the circulation [62]. Apart from the size of nanoparticles, their surface hydrophobicity determines the amount of adsorbed blood components, mainly proteins (opsonins). Many techniques have been developed and used to study the surface modification of nanoparticle bioproducts. The efficiency of surface modification can be measured either by estimating the surface charge, density of the functional groups or an increase in surface hydrophilicity. One method used to measure the surface modification is to determine zeta potential (ξ) of the aqueous suspension containing nanoparticles. It reflects the electrical potential of particles and is influenced by the composition of the particle and the medium in which it is dispersed. The main reason to measure zeta potential is to predict colloidal stability. The interactions between particles play an important role in colloidal stability. The use of zeta potential measurements to predict stability is an attempt to quantify these interactions. The zeta potential is a measure of the repulsive forces between particles. In addition, since most aqueous colloidal systems are stabilized by electrostatic repulsion, the larger the repulsive forces between particles, the less likely they will be to come close together and form an aggregate. Nanoparticles with a zeta potential above (+/-) 30 mV have been shown to be stable in suspension, as the surface charge prevents aggregation of the particles. The zeta potential can also be used to determine whether a charged active material is encapsulated within the centre of the nanocapsule or adsorbed onto the surface [63].

### 2.3 Separation and Purification of Nanoparticle Bio-products

The majority of biotechnology processes for producing pharmaceutical or diagnostic products involve the purification of proteins and peptides from a variety of sources. Those include bacteria, yeast and mammalian cell culture fluids, or extracts from naturally occurring tissue [64]. Nanoparticle (nanoparticulate) bioproducts tend to be recovered by combination of ultracentrifugation and ultra filtration protocols which are commonly scale limited, whilst dirty feedstock such as cell lysates from animal cell and microbial cultures limit the efficiency of such processes. Typically, such purification schemes contain multiple unit operations, including a number of chromatographic steps to ensure the safe removal of critical impurities and contaminants. Each step in the recovery process will affect the overall process economy by increasing operational cost and process time, and also by causing loss in product yield. Careful selection and combination of suitable unit operations during the design phase may reduce the number of steps needed [65]. Individual steps during a purification process are product specific. Typical nanoparticle bioproducts that require purification for gene therapy applications [66] include viruses, such as adenoviruses, retroviruses and adeno-associated viruses and plasmid DNA [67].

### 2.4 Loading and Release of Nanodrugs

#### 2.4.1 Drug Loading

Drug may be bound to nanoparticles either (i) by polymerization in the presence of the drug- in most cases in the form of a solution (incorporation method) or (ii) by adsorbing the drug after the formation of nanoparticles by incubating them in the drug solution. Depending on the affinity of the drug to the polymer, the drug will be surface adsorbed, dispersed in the particle polymer matrix in the form of a solid solution [68], or solid dispersion, or in some case, the drug may be covalently bound to the polymer. Therefore it is apparent that a large amount of drug can be entrapped by the incorporation method when compared to the adsorption [69]. The macromolecule or protein shows greatest loading efficiency when it is loaded at or near its isoelectric point when it has minimum solubility and maximum adsorption. The drug loading of the nanoparticles is generally defined as the amount of drug bounded per mass of polymer (usually moles of drug per mg polymer or mg drug per mg polymer) it could also be given on a percentage basis based on the polymer.

#### 2.4.2 Drug Entrapment

Binding of drug to the nanoparticle bioproducts is measured by centrifuging part of the particle suspension. For determination of drug entrapment, the amount of drug present in the clear supernatant after centrifugation is determined (*w*) by UV-spectrophotometry, fluorescence Spectrophotometer or by a validated HPLC method. A standard calibration curve of concentration versus absorbance is plotted for this purpose. The amount of drug in supernatant is then subtracted from the total amount of drug added during the formulation (*W*). Effectively, (*Ww*) will give the amount of drug entrapped in the pellet. Then percentage entrapment is given by: 

Drug entrapment (%) = , (d) pulsed delivery initiated by the application of an oscillating magnetic or sonic field [70]. In many case, some of these processes may coexist, so that the distinction between the mechanisms is not always trivial. When drug release occurs by a self diffusional process, a minimum drug loading is necessary before drug release is observed. This is easy to understand since the process involves diffusion through aqueous channels created by the phase separation and dissolution of the drug itself. This mechanism rarely occurs with drug loaded nanoparticles since, as explained before, the encapsulation efficiency of most drugs is generally too low. In fact, release from the surface and erosion or bulk polymer degradation is usually the most important processes affecting the liberation of drug from nanoparticles. Methods for quantifying drug release *in vitro *are: (i) side by side diffusion cells with artificial or biological membranes; (ii) equilibrium dialysis technique; (iii) reverse dialysis sac technique; (iv) ultracentrifugation; (v) ultrafiltration; or (vi) centrifugal ultrafiltration technique [71].

## 3 NANOPARTICLE/NANOSTRUCTURE TYPES 

Fig. (**3**) summarizes various types of nanostructures used in biomedical research and drug delivery. Below we discuss some of the important properties of these nanoparticles.

### 3.1 Inorganic Nanoparticles

Generally, inorganic nanoparticles may be engineered to evade the reticuloendothelial system by varying size and surface composition. Moreover, they may be porous, and provide a physical encasement to protect an entrapped molecular payload from degradation or denaturization. Ceramic nanoparticles are typically composed of inorganic compounds such as silica or alumina. However, the nanoparticle core is not limited to just these two materials; rather, metals, metal oxides and metal sulfides can be used to produce a myriad of nanostructures with varying size, shape, and porosity [72].

### 3.2 Polymeric Nanoparticles

Polymeric nanoparticles made of natural or artificial polymers ranging in size between 10 and 1000 nm (1 μm) [73]. Compared with other colloidal carriers, polymeric nanoparticles present a higher stability when in contact with the biological fluids. However, their polymeric nature permits the attainment of desired properties such as controlled and sustained drug release. Different approaches in the fabrication of nanoparticles consisting of biodegradable polymers have been described before [74].

### 3.3 Solid Lipid Nanoparticles

Nanoparticles made of solid lipids represent nowadays an alternative to polymeric nanoparticles. Due to their lipid nature, these nanoparticles are conceived as carriers for parenteral administration of hydrophobic drugs. However, as the potential of colloidal systems for transmucosal drug delivery has become evident, some authors have tested the utility of lipid nanoparticles for improving the oral absorption of drugs such as tobramycin, idarubicin, cyclosporine A or camptothecin. The overall conclusion from these studies is that the nanoencapsulation of these drugs in lipid nanoparticles improved their bioavailability, prolonged their blood residence time and/or modified their biodistribution [74].

### 3.4 Liposomes

Liposome is a prototype nanoparticle of semi-solid nature. Various types of liposome nanoparticles are currently used clinically as delivery systems for anticancer drugs and vaccines [75]. Liposomes are spherical lipid vesicles with a bilayered membrane structure composed of natural or synthetic amphiphilic lipid molecules [76, 77]. Liposomes have been widely used as pharmaceutical carriers in the past decade because of their unique abilities to (a) encapsulate both hydrophilic and hydrophobic therapeutic agents with high efficiency, (b) protect the encapsulated drugs from undesired effects of external conditions, (c) be functionalized with specific ligands that can target specific cells, tissues, and organs of interest, (d) be coated with inert and biocompatible polymers such as polyethylene glycol (PEG), in turn prolonging the liposome circulation half-life *in vivo*, and (e) form desired formulations with needed composition, size, surface charge, and other properties [77-79]. 

### 3.5 Nanocrystals

Nanocrystals represent an alternative to existing drug delivery technologies for poorly soluble compounds [80]. Nanocrystals are aggregates of molecules that can be combined into a crystalline form of the drug surrounded by a thin coating of surfactant. They have extensive uses in materials research, chemical engineering, and as quantum dots for biological imaging but less so in nanomedicine for drug delivery. A nanocrystalline species may be prepared from a hydrophobic compound and coated with a thin hydrophilic layer. The biological reaction to nanocrystals depends strongly on the chemical nature of this hydrophilic coating [72]. Both oral and parenteral deliveries are possible, and the limited carrier, consisting of primarily the thin coating of surfactant, may reduce potential toxicity [81]. However, the negative points are stability of nanocrystals which is limited and some therapeutic compounds may not be easily crystallized.

### 3.6 Nanotubes

Nanotubes are self-assembling sheets of atoms arranged in tubes. They may be organic or inorganic in composition and can be produced as single- or multi-walled structures. A popular version of a nanotube involves the use of soluble fullerene derivatives, such as C_60_ [72]. Carbon nanotubes (CNTs) represent an important group of nanomaterials with attractive geometrical, electrical and chemical properties and are synthesized using a variety of techniques [82]. Carbon nanotubes are generally produced by three main techniques: arc discharge, laser ablation and chemical vapour deposition. Nanotubes have large internal volumes and the external surface can be easily functionalized. While they are potentially promising for pharmaceutical applications, human tolerance of these compounds remains unknown, and toxicity reports are conflicting. It has been demonstrated that nanotubes are acutely toxic and may cause cellular death *via *an oxidative-stress pathway [83].

### 3.7 Dendrimers

Dendrimers offer a great potential for drug delivery. They are polymer-based macromolecules formed from monomeric or oligomeric units, so that each layer of branching units doubles or triples the number of peripheral groups [84]. The void area within a dendrimer, the extent of its branching, its ease of modification and preparation, and size control make dendrimers promising nanomaterials for drug delivery. Dendrimers generally have a symmetrical structure, with the potential to create an isolated ‘active site’ core area through chemical functionalization. Modification of the degree of branching may allow for encapsulation of a molecule within this structure [72].

## 4 NANODRUG DELIVERY SYSTEMS

The major goals in designing nanoparticles as a delivery system are to control particle size, surface properties [85] and release of pharmacologically active agents in order to achieve the site-specific action of the drug at the therapeutically optimal rate and dose regimen [86]. If nanoparticles are considered to be used as drug delivery vehicles, it depends on many factors including: (a) size of nanoparticles required; (b) inherent properties of the drug, e.g., aqueous solubility; (c) surface characteristics such as charge and permeability; (d) degree of biodegradability, biocompatibility and toxicity; (e) drug release profile desired; and (f) antigenicity of the final product. The advantages of using nanoparticles as a drug delivery system might be summarized as follow [87]:

1. Particle size and surface characteristics of nanoparticles can be easily manipulated to achieve both passive and active drug targeting after parenteral administration.
2. They control and sustain release of the drug during the transportation and at the site of localization, altering organ distribution of the drug and subsequent clearance of the drug so as to achieve increase in drug therapeutic efficacy and reduction in side effects.
3. Controlled release and particle degradation characteristics can be readily modulated by the choice of matrix constituents. Drug loading is relatively high and drugs can be incorporated into the systems without any chemical reaction; this is an important factor for preserving the drug activity.
4. Site-specific targeting can be achieved by attaching targeting ligands to surface of particles or use of magnetic guidance.
5. The system can be used for various routes of administration including oral, nasal, parenteral, intraocular etc.

Table **2** lists some of NPs used for delivery of drugs across the BBB.

### 4.1 HSA Nanoparticles

Albumin is an alternative macromolecular carrier and widely used to prepare microspheres and microcapsules, due to its availability in pure form and its biodegradability, nontoxicity, and nonimmunogenicity. A number of studies have shown that albumin accumulates in solid tumors making it a potential macromolecular carrier for the site-directed delivery of antitumor drugs [89]. Among these, human serum albumin (HSA) is a promising material and was used in a multitude of studies for particle preparation. HSA (molecular weight of 65 kDa) belongs to a multigene family of proteins [90] and is the major soluble protein of the circulating system with a blood concentration about 50mg ml^*−*1^. HSA consists of 585 amino acids containing 35 cysteine residues which build 17 disulfide bridges.

### 4.2 BSA-Gum Arabic Nanoparticles 

Gum Arabic (GA) is a natural composite polysaccharide rich in non-viscous soluble fiber and derived from exudates of *Acacia senegal* and *Acacia seyal* trees. GA consists of three fractions with distinct chemical structures, where the major one is a highly branched polysaccharide with a molecular weight of 3 × 10^5^ g/mol; about 10% (wt) of the total is a high molecular weight arabinogalactan-protein complex (1 × 10^6^ g/ml) and around 1% (wt) of the total contains the highest protein content (∼50 wt%) [91]. At present, GA is generally recognized as safe and has wide applications as a stabilizer, thickening agent and hydrocolloid emulsifier, mostly used in food industry, but also in the textile, pottery, lithography, cosmetics and pharmaceutical industries [92]. Several researchers are also studying the application of GA in the development of controlled drug delivery systems [93, 94] and carriers for the microencapsulation of oils and other bioactive molecules. Recently, the use of GA has been extended to the nanotechnology and nanomedicine fields, due to its biocompatibility for *in vivo* applications, as well as its stabilization of nanostructures [92].

### 4.3 α-Lactalbumin Nanoparticles

Whey proteins (WP) are important food ingredients that are used in a number of food products and drug that include dairy products, confectionary and desserts. WP composes about 20% of bovine milk and is generally produced as a co-product of the cheese industry. The utilization of WP has been an important research focus over the past few decades because of its abundance and excellent nutritional value.* α* -Lactalbumin is the major whey protein found in milk. Its molecular weight is 14176 Da and the isoelectric point is between 4.2 and 4.5. In addition, *α* -lactalbumin has significant nutritional properties and is associated with some positive health effects upon consumption. *α* -Lactalbumin has an important function in mammary secretary cells: it is one of the two components of the lactose biosynthesis in the lactating mammary gland. Its amino acid composition seems to be optimal for the requirements of the infant and it has a high digestibility [92]. It is relatively rich in tryptophan (four residues per molecule) and *α*-lactalbumin consumption increase plasma tryptophan which is known to have a positive effect on satiety and mood. However, *α* -lactalbumin consumption was shown to improve mood and cognitive performance in vulnerable subjects. *α*-lactalbumin itself (or its fragments) possesses bacterial or antitumor activity. It can be used as a basis for design of antitumour agent, acting through disorganization of chromatin structure due to electrostatic interaction between *α*-lactalbumin and histone proteins [92]. 

Therefore, whey proteins have interesting functional property and can be used as nanoparticles systems for encapsulation and controlled delivery application. Since *α* -lactalbumin is a milk protein, it will be fairly easy to apply in foods and pharmaceutics. It has been demonstrated that *α* -lactalbumin the second most abundant protein in the whey fraction of bovine milk, can be induced to form nanoparticles [92].

### 4.4 Gelatin Nanoparticles

Gelatin is a naturally occurring polymer with relatively low antigenicity and has been used for decades in parenteral formulations and as an approved plasma expander [95]. In addition, its biodegradability, biocompatibility, non-toxicity, ease of chemical modification and cross-linking make gelatin-based nanoparticles a promising carrier system for drug delivery [96]. In a report in the field of immunotherapy, gelatin nanoparticles have been utilized to target CpG oligonucleotides to the lymph node in order to elicit a more efficient antitumoral immunity [97]. In addition, cationized gelatinnanoparticles have been found to strongly increase the immunostimulatory effects of CpG oligonucleotides [98]. The primary structure of gelatin offers many possibilities for chemical modification and covalent drug attachment. This can be done either within the matrix of the particles or only on the surface of the particles. In the former case, chemical modifications have to be done to the gelatin macromolecules before nanoparticles are formed, while in the latter case, the surface of the particles is used [99]. 

## 5 NERVOUS SYSTEM NANODRUGS

Nanomaterials and nanoparticles can interact with biological systems at fundamental and molecular levels [100, 101]. In neuroscience, the application of nanotechnologies entails specific interactions with neurons and glial cells. Nanodevices can target the cells and glia with a high degree of specificity. This unique molecular specificity enables the nanodrugs to stimulate and interact with tissues and neurons in controlled ways, while minimizing undesirable effects. There are two main types of nervous system drugs (neurodrugs): behavioural and molecular. Behavioural neurodrugs are for the study of how different drugs affect human behaviour and human brain. These drugs are usually used for diagnosis and therapy of neurodegeneration disorders [47, 102]. Molecular neurodrugs are used for the study of neurons and their neurochemical interactions. Since for the most part, neurons in the human brain communicate with one another by releasing chemical messengers called neurotransmitters, these drugs have to target specific transmitters and receptors to have beneficial effect on neurological functions. The preparation of these two types of drugs is closely connected. Researchers are studying the interactions of different neurotransmitters [103], neurohormones [104], neuromodulators [105], enzymes [106], second messengers [107], co-transporters [108, 109], ion channels [110], and receptor proteins [111] in the central and peripheral nervous systems to develop drugs to treat many different neurological disorders, including pain [112], neurodegenerative diseases such as Parkinson's disease [113] and Alzheimer's disease [114], psychological disorders [115], addiction [116], and many others.

The blood–brain barrier significantly hinders the passage of systemically delivered therapeutics and the brain extracellular matrix limits the distribution and longevity of locally delivered agents. Nanoparticles represent a promising solution to these problems. They can cross blood-brain barrier and enter the CNS. Although the applications of nanotechnology in basic and clinical neuroscience are only in the early stages of development, partly because of the complexities associated with interacting with neural cells and the mammalian nervous system, however the early results show an impressive potential of nanotechnologies to contribute to neuroscience research [117]. One area in which nanotechnology may have a significant clinical impact in neuroscience is the selective transport and delivery of drugs and other small molecules across the blood brain barrier that cannot cross otherwise.

Examples of current research include technologies that are designed to better interact with neural cells, advanced molecular imaging technologies [118, 119], materials and hybrid molecules used in neural regeneration [120], neuroprotection [121], and targeted delivery of drugs and small molecules across the blood–brain barrier [122, 123]. Among all these modern methods of drug delivery to the central nervous system (CNS), the design and application of bionanotechnologies aimed at the CNS provide revolutionary new approaches for studying cell and molecular biology and physiology. The successful and meaningful development of bionanotechnologies designed to interact with the CNS as research or clinical tools require an understanding of the relevant neurophysiology and neuropathology, an appreciation of the inherent 'nanoscale' structure of the CNS, and an understanding of the relevant chemistry and materials science and engineering. At nanoscale, consideration of individual molecules and interacting groups of molecules in relation to the bulk macroscopic properties of the material or device becomes important, since it is control over the fundamental molecular structure that allows control over the macroscopic chemical and physical properties [124]. Applications to neuroscience and physiology imply materials and devices designed to interact with the body at subcellular (i.e., molecular) scales with a high degree of specificity. This can potentially translate into targeted cellular and tissue-specific clinical applications designed to achieve maximal therapeutic affects with minimal side effects.

It started with controlled release strategy and the development of miniaturized delivery systems [125] and continued by the application of albumin nanoparticles for the first time in the Johns Hopkins Medical Institution in Baltimore [126]. Other nanoconstructs such as drug-polymer conjugates were first proposed in the 1970s [127] and developed pre-clinically in the 1980s [128]. Prof. Kreuter [129] proposed a definition of polymeric nanoparticles for pharmaceutical purposes for the first time that later was adopted by the Encyclopaedia of Pharmaceutical Technology [130] and the Encyclopedia of nanotechnology [131]. Today, more than 25 nanomedicines have already been approved for human use [102]. Usually the application of nanodrugs to neuroscience is divided into two parts: application in basic neuroscience [124], and in clinical neuroscience [27]. 

The development of nanotechnology products may play an important role in adding a new group of therapeutics to the products of pharmaceutical companies [132]. Nanotechnology enhances (1) delivery of poorly water-soluble drugs; (2) delivery of large macromolecule drugs to intracellular sites of action; (3) targeted delivery of drugs in a cell- or tissue-specific manner; (4) transcytosis of drugs across tight epithelial and endothelial barriers; (5) co-delivery of two or more drugs or therapeutic modality for combination therapy; (6) visualization of sites of drug delivery by combining therapeutic agents with imaging modalities; and (7) real-time read on the *in vivo *efficacy of a therapeutic agent [133]. Additionally, the manufacturing complexity of nanotechnology therapeutics may also create a significant hurdle for generic drug companies to develop equivalent therapeutics readily [132]. 

To develop effective nanoparticle-based delivery and diagnostic systems it is essential to understand how the human body clears particles [134, 135]. A review by Emerich and Thanos discussed some important mechanisms in which nanoparticles have advantages for drug delivery [136]. Here we give a brief overview of these mechanisms and nanoparticle advantages. The body distributes nutrients, clears waste, and distributes systemically administered drugs *via *the vascular and lymphatic systems. At first glance this intricate system seems to preclude any chance of specific, compartmentalized drug targeting. Intravenously injected particles are scavenged and cleared from the circulation by Kupffer cells and macrophages in a process that is facilitated by surface deposition of blood opsonic factors and complement proteins on the injected drug particle [137]. Some *in vivo* studies have provided detailed information regarding the phagocytosis and clearance of carbon nanotubes *in vitro* [138, 139]. Both clearance and opsonization can, however, be influenced by the size and surface characteristics of injected particles. Relative to very small particles, particles 200 nm or greater in diameter activate the complement system more efficiently and are cleared faster from the circulatory system. Although the biological mechanisms underlying this phenomenon are poorly understood they are likely related to the basic geometry and surface characteristics (charge, functional groups, etc.) of particles that mediate binding of blood proteins and opsonins [140-142]. In principle, differential opsonization and clearance may be favorably modulated using nanoparticles with engineering characteristics tailored to the phenotype, physiological activity, and recognition mechanisms of specific subpopulations of macrophages [143]. Likewise, nanomaterials less than 100 nm are associated with variable levels of potential toxicity albeit by different mechanisms. For example, inhaled particles can elicit pulmonary inflammation and oxidative stress [144] and disrupt distal organ function. As nanoparticles decrease in size, their relative surface area in air is increased, causing an enhanced exposure to several proposed toxic mechanisms including hydrophobic interactions, redox cycling, and free radical formation. In this manner, a 20 nm particle would have roughly 100 times the inherent toxicity of a 2 μm particle in an equivalent dose based on mass, assuming a direct relationship with surface area.

The surface architecture and chemistry of nanoparticles also impacts the pharmacokinetic profile of a given drug by altering its aggregation in the body [134, 135]. Small diameter particles have a large relative surface area that is inherently prone to aggregation. While nanoscale objects including dendrimers, quantum dots, and micelles are prone to aggregation this phenomenon can be significantly obviated using surface engineering. Quantum dots can be made soluble, dispersible, and stable in serum by coating their surface with polydentate phosphine or hydrophilic polymers [145, 146]. Alternatively, the surface of nanoparticles can be manufactured with macromolecular fibers made from polymers such as poly(ethyleneglycol) [142, 143], that suppress macrophage recognition by reducing protein adsorption and surface opsonization. The evasion of macrophages and suppression of opsonization prolongs the circulation of nanoparticles allowing for the controlled release of therapeutics in the blood. In cases such as intravenously injected radiolabelled carbon nanotubes it appears that the nanotubes are not retained by the liver or spleen but are rapidly cleared (half-life of 3 h) intact from the blood by renal excretion [139].

Controlling the pharmacokinetic profile of circulating particles also provides the basis for beginning to develop approaches that confine injected particles to the vascular system by preventing leakage from the vasculature or avoiding splenic filtration. This is particularly useful in pathological settings such as cancer where the tumor-associated blood vessels are a potential therapeutic target [147]. Recent developments are revealing the molecular signatures within endothelial cells and the vascular and lymphatic beds allowing systems to be designed to target therapeutics and diagnostics directly to specific pathological vessels. Successful nanoparticle design requires assembly of the appropriate targeting ligands on nanocarriers and long circulating nanosystems with appropriately engineered surface curvature and reactivity to control particle stability, aggregation, receptor binding and subsequent biochemical cascades and signaling processes. Sometimes, however, the preferred method of nanoparticle delivery may be direct interstitial injection. The fate of interstitially injected nanoparticles is also dependent on the size and surface characteristics of the injected particle [137, 148]. The size of the particles must be large enough (30–100 nm) to avoid leakage into blood capillaries but not so large (>100 nm) that they become susceptible to macrophage-based clearance. Surface manipulation can control the extent of particle aggregation at interstitial sites, optimizing/controlling drainage kinetics and lymph node retention [137]. For instance, hydrophilic, but not hydrophobic nanoparticles, repulse each other and interact poorly with the ground substance of the interstitium and drain rapidly into the initial lymphatics.

Very small particles (1–20 nm) with long circulatory residence times slowly extravasate from the vasculature into the interstitial spaces to be transported by lymphatic vessels to lymph nodes [149]. This phenomenon is quite important when designing nanoparticledrug carriers as the extent of nodal vascularization and blood supply varies across tissues allowing differential leakage from the blood pool through the permeable endothelium in lymph nodes. These modes of particle movement from the blood and interstitial sites to the lymph nodes provide opportunities for diagnostic imaging where, essentially, only an enhancement of signal over background is required. The physicochemical properties of quantum dots [150] and superparamagnetic iron oxide nanocrystals [151] make them ideal for such purposes.

### 5.1 Nervous System Nanodrug Delivery for Research

The central nervous system directs the functions of all tissues of the body. Chemical influences are capable of producing a myriad of effects on the activity and function of the central nervous system. Since our knowledge of different regions of brain function and the neurotransmitters in the brain is limited, the explanations for the mechanisms of drug action may be vague. The fundamental functional scale of the central nervous system is at the micro and nanoscales. The structure and organization of the CNS starts from macro levels down to the protein (i.e. nanoscale) level. The study of nanostructure of the CNS can provide motivation for the potential nanotechnology has to offer neuroscience and the development of new treatments for neurological disorders. Basic research applications of neuronanodrug primarily concern themselves with understanding basic molecular and cellular mechanisms without necessarily considering their potential clinical implications, whereas clinical neuronanodrug applications are designed to primarily target disease events, and make use of basic molecular and cellular neurobiology only when necessary.

Applications of nanotechnology for basic research in neuroscience includes three specific areas: (a) nanoengineered materials and approaches for promoting neuronal adhesion and growth to understand the underlying neuro biology of these processes or to support other technologies designed to interact with neurons *in vivo *(for example, coating of recording or stimulating electrodes) [100, 152-154]; (b) nanoengineered materials and approaches for directly interacting, recording and/or stimulating neurons at a molecular level [155, 156]; and (c) imaging applications using nanotechnology tools, in particular, those that focus on chemically functionalized semiconductor quantum dots [157-159].

Studying molecular details of synapses are particularly important since they are examples of naturally occurring form of a nanoengineered structure [160, 161]. The neuronal synapse, both presynaptically and postsynaptically, is an amazing nanoscale machine of exquisite complexity and specialization [162, 163]. The synapse is made up of pre and postsynaptic membranes in direct opposition to each other separated by a very narrow gap of about 25 nm. Across this gap extend projections from both the presynaptic and postsynaptic cells that hold the synapse tightly together, so much so that it is difficult to physically separate presynaptic and postsynaptic terminals. On the postsynaptic side is the postsynaptic density (PSD), an ultrastructurally dense collection of intracellular structures, proteins, and vesicles that can be easily seen with electron microscopy [164]. The action potential and synapse constitute the fundamental mechanisms by which information is transmitted through the nervous system. Because a single astrocyte can have multiple processes associated with many neurons, neuronal-glial signaling adds a significant layer of computational complexity to information processing and flow in the CNS since these cells are able to ‘short circuit’ neuronal connections. This is one of the most important and active areas of present day molecular neuroscience and its implications will most likely impact on the way to approach neurological disorders at a molecular level. It also provides opportunities to study these mechanisms using nanoengineered approaches such as quantum dots, patterned culture systems with nanoscale features, and high throughput electrophysiology. The fundamental functional units that make up the cell are proteins and other molecules, the principal targets which nanoengineered materials and devices are designed to interact with [165]. This can be achieved in one of two ways: One is to develop bionanotechnologies that target ubiquitous components of cellular and biochemical signaling systems. Depending on the specific terminal phenotype with which the device interacts, targeting a ubiquitous signaling pathway would affect one or more specific downstream events. A good example would be targeting protein phosphorylation sites, which is a ubiquitous mechanism for modifying and altering the function of proteins. Targeting phosphorylation sites on b-tubulin III would produce specific effects in neurons, since it is a neuron specific microtubule, while doing the same thing on glial fibrillary acidic protein (GFAP) phosphorylation sites in astrocytes, a macroglial specific intermediate filament, would produce very different results. The second example would be to develop bionanotechnologies designed to target a cell-specific signaling process in order to affect a known functional end point. A particularly significant potential target for nanotechnologies designed to interact with neurons, for example, is the laminin family of proteins [166, 167]. The laminins have been used in micro- and nanoengineered systems with neurons both *in vitro *and *in vivo *particularly within the context of neural regeneration. Laminins are large multi-domain trimeric proteins made up of a, b, and g chains, of which there exist various isotypes due to proteolytic cleavage and alternative splicing posttranslational modifications [168, 169]. In fact all cells, in a very real sense, are exquisitely engineered nanoscale machines designed to carry out many complex tasks in a coordinated way that respond, signal, and adapt to the environment in which they find themselves.

In part due to the inherent complexity of the CNS and in part due to its difficult and restricted anatomical access, nanotechnology applications aimed at the CNS are lagging behind other areas of medicine and biology, such as orthopedic applications, DNA/genomic sensors, and novel drug and gene delivery approaches. The CNS represents an extremely heterogeneous cellular and molecular environment, with many different types and sub-types of cells. This degree of cellular heterogeneity underlies the nervous system’s anatomical and functional ‘wiring’ that is the basis of the system’s extremely complex information processing. Nanotechnologies designed to interact with CNS cells and processes *in vivo *must take this complexity into consideration, even if they are designed to target a specific cell type or physiological process. Failure to do so may result in unforeseen ‘side effects’ or interactions. In general, lipid soluble factors are able to cross the BBB much more readily than less lipid soluble factors, a fact that has been a central theme for drug delivery into the CNS and a potential target for nanotechnological approaches to do the same [170]. In addition the mechanisms of many nanodrugs by which they cross the BBB is not fully understood. Considerable amount of research is being done to investigate the mechanisms of nanodrug deliveries to the CNS. Cellular uptake mechanisms vary according to cell type, physicochemical properties of the internalized compound and the mechanisms of activation of the drug or cell carrier [171-173]. The more we know about and understand how the healthy CNS works and how it fails in disease, the better we can design and apply bionanotechnologies that are highly specific to one or more CNS processes.

Numerous transport mechanisms define the BBB. Small lipophilic molecules most easily pass from the capillaries. Those molecules that are charge bearing, large or hydrophilic, require gated channels, ATP, proteins and/or receptors, to facilitate passage through the BBB. One exceptional regulatory aspect of the BBB is that it is not fully present throughout the brain. The circumventricular organs, which border the ventricles of the brain, do not possess a BBB [174]. Transport mechanisms at the BBB can be manipulated for cerebral drug targeting. Variable efficiencies of endocytosis mechanisms, intracellular trafficking, release of the therapeutic agent into the cytoplasm, diffusion and translocation of the therapeutic agent to its susceptible target, and partition into the nucleus or other organelles alter the actual activity of the therapeutic agent [72]. Nanoparticles present an interesting opportunity for eliminating much of this ‘waste’ due to masking of the therapeutic agent from its biological environment; this effectively limits the influence of a compound’s physical properties on intracellular drug concentrations. Instead, the properties and surface characteristics of the nanoparticle play a greater role in compound delivery and resulting intracellular drug concentrations. To improve the therapeutic potential of the nanoparticle-based carriers for the intracellular delivery, it is important to understand the physicochemical properties of nanoparticles affecting the cellular uptakemechanism and the intracellular trafficking. Table **3** is listing a number of important nanoparticle-based drugs for research and preclinical trials. Naturally, ideal drug candidates should be small, lipophilic (as measured by the octanol: water partition coefficient), hydrophobic, and compact (a parameter measured by the polar surface area). Polymeric nanoparticles are advantageous in a number of ways.

For one, they possess the high drug-loading capacities, thereby increasing intracellular delivery of the drug. Secondly, the solid matrix of particulate carriers protects the incorporated drugs against degradation, thus increasing the chances of the drug reaching the brain. Furthermore, carriers can target delivery of drugs, and this targeted delivery can be controlled. One additional benefit of particulate carriers is that their surface properties can be manipulated in such a way as to evade recognition by the macrophages of the reticuloendothelial system (RES), hence improving the likelihood of nanoparticles reaching the brain [174]. Rational surface modification and coatings of nanoparticles can modulate pharmacokinetic properties (e.g., blood half-life, elimination and biodegradation), toxicity, immunogenicity and efficient targeting. Targeting has generally been achieved by conjugating nanoparticle surfaces to antibodies. For example it has been shown that cell uptake and tumor retention of folate-coated nanoparticles was significantly enhanced over PEG-coated gadolinium (Gd) nanoparticles [186]. Also It has been demonstrated that size plays a role in cellular uptake of liposomes, polymer NPs, artificial viruses (DNAcoated glycocluster NPs), and inorganic nanostructures [187]. Researchers have studied the relationship between both NP size and shape (of the same material composition) and its uptake kinetics and mechanism. It was shown that NPs with a diameter of ~50 nm were taken up by mammalian cells at a rate and concentration that was faster and higher, respectively, than other sizes and shapes [188]. Detailed studies of uptake kinetics of nanoparticles by cells have not been well characterized and quantified as a function of their size and shape (i.e., trends have not been determined). Most studies have focused on liposomes [189, 190] and polymer particles [191, 192] which are generally larger than 100 nm. To date, several reports have discussed the internalization of polymeric nanoparticles into the cells by endocytic pathways. Endocytosis is a conserved process in eukaryotes where extracellular substances are taken up into the cells usually by the invagination of plasma membrane forming vesicles [193]. Furthermore, metallic, semiconductor, and carbon-based nanoparticles can be synthesized with greater size and shape variabilities than liposome and polymer particles. Superparamagnetic iron oxide is another type of nanoparticles that have been recognised as a promising tool for the site-specific delivery of drugs and diagnostics agents [194]. Magnetic properties and internalisation of particles in cells depend strongly on the size of the magnetic particles. Particles below 100 nm are small enough both to evade reticuloendothelial system (RES) of the body as well as penetrate the very small capillaries within the body tissues [195, 196]. Because of their hydrophobic surfaces and large surface area to volume ratio, *in vivo* use of nanoparticles is hampered by very rapid clearance of nanoparticles from the circulation by the RES. Avoidance of this obstacle is possible if the surfaces of these nanoparticles are made sufficiently hydrophilic, as these modifications prolong considerably the nanoparticle half-life in the circulation [197]. Although this approach has succeeded for *in vitro* sensing, its *in vivo* application has proved more challenging because of cost, limited shelf life, regulatory hurdles and potential immunogenicity after repeat injections of such preparations. Another targeting approach with promising initial results involves conjugation of nanoparticles to peptides but synthetic costs can be high [198]. 

Nanodrug delivery is an interdisciplinary and independent field of research and is gaining the attention of pharmaceutical researchers, medical doctors and industry. Ideally if the nanodelivery-drug complex were to be administered systemically (e.g., intravenously) it would need to find the CNS while exhibiting minimal systemic effects, be able to cross the BBB, target whatever it needs to target once in the CNS, and only then carry out its primary active function such as the release of the drug. These are technically very demanding challenges that require multidisciplinary interactions between different fields including engineering, chemistry, cell biology, physiology, pharmacology, medicine, and others.

A safe and targeted drug delivery could improve the performance of some classic medicines already on the market, and moreover, will have implications for the development and success of new therapeutic strategies such as anticancer drug delivery, peptide and protein delivery and gene therapy. In the last decade, several drug-delivery technologies have emerged and a fascinating part of this field is the development of nanoscale drug delivery devices. The design and application of bionanotechnologies aimed at the central nervous system (CNS) provide powerful new approaches for studying cell and molecular biology and physiology. Nanoparticles have been developed as an important strategy to deliver conventional drugs, recombinant proteins, vaccines and more recently, nucleotides.

Carbon nanotubes (CNTs) represent an important group of nanomaterials with attractive geometrical, electrical and chemical properties and are synthesized using a variety of techniques [199]. CNTs are carbon vapor grown, self-assembled from peptide amphiphiles or electrospun from most polymer materials. Carbon nanotubes have attracted attention in nanomedicine although there are also serious concerns regarding their safety. To integrate CNTs into biological systems, CNTs need to be functionalized. Functionalization can make CNTs soluble and improve their biocompatibility properties. In addition, through functionalization, bioactive agents can be conjugated to CNTs which can serve as a carrier for drugs, antigens and gene delivery. The ability of CNTs to cross cell membranes has allowed them to become of particular high interest for drug delivery strategies [200]. 

More recently some novel experimental neuroprotective nanomaterials, such as fullerenes were introduced to the neuropharmacology which have antioxidant properties to eliminate reactive oxygen species in the brain to mitigate oxidative stress [201].

### 5.2 Nervous System Nanodrug Delivery for Diagnosis and Therapy

Any nervous system drug must cross the blood-brain barrier to penetrate the CNS and have its effect on the target site. Applications of nanotechnology that are intended to limit and reverse neurological disorders by promoting neural regeneration and achieving neuroprotection are active areas of clinical neuronanotechnological research [100]. Although all three of the basic nanotechnology applications can also contribute to an understanding of neuropathophysiology, however, for the most part, applications of nanotechnology in clinical neuroscience primarily concern themselves with limiting and reversing neuropathological disease states. Generally, the clinical applications of nanotechnology in neuroscience include (a) nanotechnology approaches designed to support and/or promote the functional regeneration of the nervous system [202]; (b) neuroprotective strategies, in particular those that use fullerene derivatives [203-206]; and (c) nanotechnology approaches that facilitate the delivery of drugs and small molecules across the BBB [207-212]. One of the frontiers of neuropharmacotherapy is the delivery of drugs to the central nervous system (CNS) with a high degree of specificity and enough concentration. To a large extent this is because of the inability of many candidate therapeutic drugs to readily cross the blood–brain barrier (BBB) and reach sufficiently high concentrations at sites of action within the brain [213]. Novel technologies to enable nanomaterials to cross the blood brain barrier will allow efficient systemic delivery of therapeutic and diagnostic agents to the brain.

Numerous disorders of the central nervous system (CNS) has been identified which are usually severe and affect a large portion of the world’s population [27]. In neurodegenerative diseases, such as Alzheimer’s diseases (AD), Parkinson’s diseases (PD) and multiple sclerosis, patients experience symptoms related to movement, memory, and dementia due to the gradual loss of neurons. Common chronic neurological disorders like epilepsy, migraine, chronic pain and psychiatric disorders such as anxiety, depression, and schizophrenia are debilitating conditions that significantly affect the morbidity and mortality of modern society. Brain tumors constitute a profound and unsolved clinical problem and are a common cause of cancer-related death especially for children. The causes of CNS disorders are complex and associated with many factors such as advancing age, environmental cues, and disordered immunity and less with the host genetics [214]. Diagnostic decisions for neurologic and neurodegenerative disorders can often be clouded by concomitant depression, motor impairments, and lethargy that follow debilitating immune suppression and weight loss. In reality, cognitive, motor, and behavior abnormalities underlie a variety of neurological dysfunctions associated with neurodegenerative disorders and HIV-1 infection [47]. Thus, combinations of clinical, laboratory, and neuroimaging tests, while not irrefutably conclusive, are essential to provide diagnostic support [215-217]. A wide range of neuroimaging techniques can aid in the diagnosis of neurological complications associated with neurodegenerative disorders. These techniques include nuclear medicine studies such as positron emission tomography (PET) and single photon emission computed tomography (SPECT). Morphological studies include magnetic resonance imaging (MRI) and computed tomography (CT). Studies of brain physiology and biochemistry consist of SPECT and MRI perfusion, magnetization transfer MRI (to assess myelin loss), ^1^H and ^31^P magnetic resonance spectroscopy (MRS) and magnetic resonance spectroscopic imaging (MRSI) [218, 219]. These techniques have the potential to identify underlying neurological processes involved in disease progression. Thus, MRI, CT, or diffusion tensor imaging (DTI) is often used to supplement clinical and neurological examinations for diagnosis of a variety of degenerative disorders of the CNS including HIV-1 associated cognitive impairments [220, 221]. These radiographic and functional imaging tests can delineate the structural and metabolic effects of diseases of the brain and differentiate them from other types of degenerative, infectious or cancerous lesions. CT, MRI, and DTI easily depict brain atrophy. Computerized tomography of the brain characteristically can show mass lesions or cerebral atrophy and is reflective of moderate to severe disease [222, 223]. 

Safe, site-specific, and efficient delivery of compounds to CNS disease sites remains a singular goal in achieving optimal therapeutic outcomes to combat neurodegenerative diseases. Treatment of CNS disorders by systemic administration or local delivery of drugs is currently inefficient in many cases. Furthermore, clinical neuroscience faces great challenges due to the extremely heterogeneous cellular and molecular environment and the complexities of the brain’s anatomical and functional “wiring” and associated information processing [224]. However, the emergence of nanotechnology provides hope that it will revolutionize diagnosis and treatment of CNS disorders. Neurodegenerative diseases are usually linked to a loss of brain and spinal cord cells. For example, the neuronal damage in AD and PD is associated with abnormal protein processing and accumulation and results in gradual cognitive and motor deterioration [225]. One promising strategy for treatment of neurodegenerative diseases is to support and promote neurite and axonal growth by implanting nanometer-scale scaffolds using tissue-engineering approaches. Some promising nanomaterials to use for this application are nanotubes [28, 29] and nanofibers [226]. These materials mimic tubular structures that appear in nature, such as rod shaped bacteria or viruses, microtubules, ion channels, as well as axons and dendrites. It has been shown that polymeric nanoparticles are good candidates to deliver drugs to the CNS. 

Polymeric nanoparticles can be engineered for effective systemic and local delivery of therapeutics to the CNS. Moreover, many of the polymers used in nanoparticle fabrication are both biodegradable and biocompatible, thereby increasing the clinical utility of this strategy [227]. Some of the different types of polymeric nanoparticles were discussed above. Nanomedicine approaches have revolutionized the treatment of cancer and vascular diseases, where the limitations of rapid nonspecific clearance, poor biodistribution and harmful side effects associated with direct systemic drug administration can be overcome by packaging the agents within sterically stabilized, long-circulating nanovehicles that can be further surface-modified with ligands to actively target cellular/molecular components of the disease [228]. The need for the discovery of new diagnostic and therapeutic agents effective against central nervous system diseases is increasing every day. Here we provide an overview of novel nanomaterials that have potential to improve diagnosis and therapy of neurodegenerative disorders. Examples include liposomes, nanoparticles, polymeric micelles, block ionomer complexes, nanogels, and dendrimers that have been tested clinically or in experimental models for delivery of drugs, genes, and imaging agents. Table **4** lists some of clinically approved nanoparticle-based therapeutics for nervous system related diseases. Liposomes are vesicular structures composed of unilamellar or multi-lamellar lipid bilayers surrounding internal aqueous compartments. Their sizes vary from several nanometers to several microns.

Relatively large amounts of drug molecules can be incorporated into liposome aqueous compartments (water soluble compounds) or within lipid bilayers (lipophilic compounds). Conventional liposomes usually are rapidly cleared from circulation by reticuloendothelial system (RES). Extended circulation time can be accomplished with small sized liposomes (<10 nm) composed of neutral, saturated phospholipids and cholesterol. Furthermore, many modern studies use liposomes with a surface modified with polyethylene glycol (PEG) [47]. Liposomes are readily taken up by macrophages, microglia and astrocytes in the CNS. PEGylated liposomes accumulate more rapidly in brain when the BBB is compromised such as in experimental autoimmune encephalomyelitis (EAE) [229]. Interestingly, brain accumulation of liposomes labeled with radioactive isotope, ^99m^Tc is increased during EAE [230]. Incapable of penetrating a normal BBB, immunoliposomes used to treat glial brain tumors that express GFAP reach their disease site when the BBB is partially permeabilized. Liposomes have also been conjugated with mannose, transferrin and insulin receptors at the surface of brain capillaries. In addition considerable work was reported on antibodies to transferrin receptor, OX26, that were linked to the surface of PEGylated liposomes *via *PEG spacers. Such immunoliposome constructs were used to deliver small drugs, Daunomycin and Digoxin as well as plasmid DNA to the brain [47]. 

Nanoparticle is another nanocarrier for CNS drug delivery [231]. They are often composed of insoluble polymer(s) [227, 232]. During their formulation drug is captured within the precipitating polymer, forming nanoparticle, and then released upon degradation of a polymer in the biological environment. The methods for preparation of nanoparticles commonly employ the use of organic solvents that may result in degradation of immobilized drug agents; especially biomacromolecules (see section 2). The size of nanoparticle should not exceed 100-200 nm to allow for efficient uptake. Poly(butylcyanoacrylate) is one of typical nanoparticles that were evaluated for CNS delivery of several drugs [231, 233]. Usually PEG-containing surfactants, such as Tween 80 are used to coat these nanoparticles. After injection, they localized in the choroid plexus, *via *mater and ventricles, and, to a lower extent, in the capillary endothelial cells [47]. Some examples of drugs delivered to CNS in these constructs are, analgesics (Dalargin, Loperamide), anti-cancer agents (Doxorubicin), anti-convulsants (NMDA receptor antagonist, MRZ 2/576), and peptides. For example, nanoparticles prolonged anti-convulsive activity of MRZ 2/576 compared to the free drug. Recently nanoparticles conjugated with metal chelators, Desferioxamine or D-Penicillamine, were shown to cross the BBB, chelate metals, and exit through the BBB with their complexed metal ions. This method may prove to be useful for reducing the metal load in neural tissue thus mitigating harmful effects of oxidative damage during AD and other CNS diseases. Nanospheres, a subset of nanoparticles, are hollow species prepared by microemulsion polymerization or covering colloidal templates with a thin layer of polymer material followed by template removal. Carboxylated polystyrene nanospheres (20 nm) were evaluated for CNS drug delivery. After intravenous injection such nanospheres remained in the vasculature under normal conditions. However, they extravasated into brain during cerebral ischemia-induced stress that partially opened the BBB [234]. Such nanospheres have potential for CNS delivery of drugs and imaging agents during ischemia, stroke and other conditions that disrupt the BBB. Nanosuspensions are crystalline drug often stabilized by non-ionic PEG-containing surfactants or with mixtures of lipids [235, 236]. Table **5** lists some of the nanoparticle-based nervous system related therapeutics in clinical trials.

Another type of nanoparticles for the CNS drug delivery are polymeric micelles (‘‘micellar nanocontainers’’) which have been developed as drug carriers [237] and diagnostic imaging agents. They usually have sizes from 10 to 100 nm. The core of polymeric micelles can incorporate considerable amounts (up to 20–30%wt) of water-insoluble drugs preventing premature drug release and degradation. The shell stabilizes micelles in dispersion and masks the drug from interactions with serum proteins and untargeted cells. After reaching target cells drug is released from the micelle *via *diffusion. Several clinical trials are completed or underway to evaluate polymeric micelles for delivery of anti-cancer drugs. For example micelles of Pluronics block copolymers (PEG-b-PPG-b-PEG) were used as carriers for CNS drug delivery [238].

Polyion complex micelles (also called ‘‘block ionomer complexes’’) are also novel nanosystems for incorporation of charged molecules. The core contains polyion complexes of a biomacromolecule and ionic block of the copolymer. The shell is formed by the non-ionic block. In case of surfactant- based complexes the core is composed of mutually neutralized surfactant ions and polyion chains. These nanomaterials were shown to efficiently deliver DNA molecules *in vitro* and *in vivo*. Although no study on their delivery to CNS was reported so far, however it has a potential to be used as CNS drug delivery nanoparticle. 

Nanosized networks of cross-linked polymers are called nanogels which often combine ionic and non-ionic chains like polyethyleneimine (PEI) and PEG. Nanogels swell in water and incorporate through ionic interactions of oppositely charged molecules such as oligonucleotides, siRNA, DNA, proteins and low molecular mass drugs. The advantage of nanogels for CNS drug delivery is that they can load with very high capacities (up to 40-60% wt) which cannot be achieved with conventional nanoparticles. Individual collapsed nanogels particles do not phase separate and form stable dispersions because of solubility of PEG chains. *In vivo* studies suggested that nanogels increased uptake of oligonucleotides while decreasing its uptake in liver and spleen. Overall, nanogels are promising carriers for CNS drug delivery, although they are in relatively early stages of development [47].

Dendrimers are another type of nanoparticle which is regularly branched polymer molecules with branches growing from one or several centers. They can be formulated nonocovalently with biological agents, such as DNA or conjugated with pro-drug or imaging agents and thus can be used as delivery vehicles for drug therapy or molecular imaging. It has been reported that dendrimers fulfill the requirements as carriers for gene, nucleic acids, bioactive molecules and peptide/protein delivery aimed at modulate the cells functions, *in vitro* and *in vivo* [239]. This novel class of polymers and their derivatives exhibit unique physicochemical and biological properties, which have great potential for use in a variety of applications, including tissue engineering and regenerative medicine [240].

**Fig. (4):**
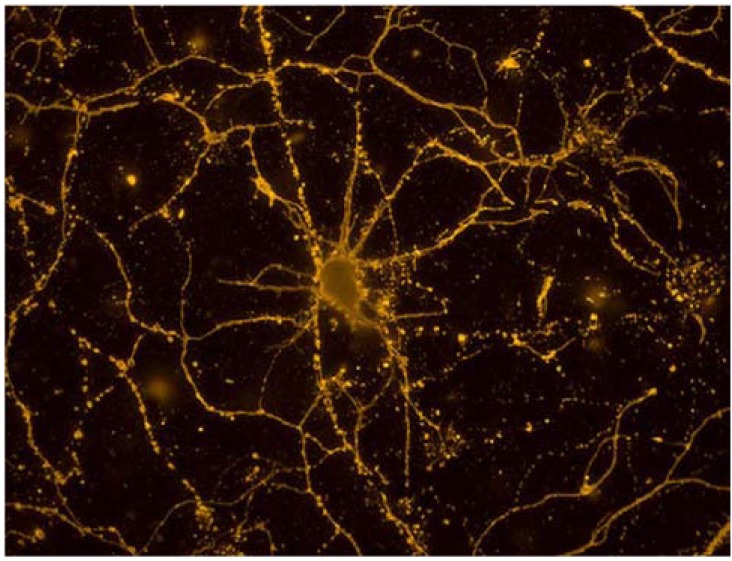
Primary cortical neurons labeled with beta-tubulin III chemically functionalized 506 nm quantum dots (beta-tubulin is a neuronal specific intermediate filament protein) (provided by Dr. Gabriel Silva, UCSD; adapted from [136]).

Application of nanoparticle technology has improved some of diagnostic and screening techniques. One of the first applications of nanoparticle technology is improved fluorescent markers [136]. While fluorescent markers are routinely used in basic research and clinical diagnostic applications, there are several inherent disadvantages with current techniques, including the requirement for colormatched lasers, the fading of fluorescence even after a single use and the lack of discriminatory capacity of multiple dyes due to the tendency of the different dyes to bleed together. Fluorescent nanoparticles potentially overcome these issues. One iteration of this technology uses quantum dot nanocrystals (e.g. colloidal inorganic semiconductor nanocrystals consisting of a CdSe core and a ZnS shell) that are several nanometers in diameter and manufacturable in a nearly unlimited range of sharply defined colors [241]. Aside from the clinical potential of these particles, their research value includes the possibility of simultaneously tagging multiple biomolecules both on and inside cells to monitor the complex cellular changes and events associated with disease, providing valuable clues for the development of future pharmaceuticals and therapeutics. A particularly interesting approach is being taken by Silva (2006) where quantum dot nanocrystals are being used to provide superior resolution of intracellular distribution of cytoskeletal components during normal and pathological central nervous system events (see Fig. **4**).

A related technology called Probes Encapsulated by Biologically Localized Embedding (PEBBLES), pioneered by Raoul Kopelman, allows dye tagged nanoparticles to be inserted into living cells to monitor metabolism or disease conditions [242, 243]. This system was used to quantify zinc levels within living cells. A similar application uses superparamagnetic nanoparticles as magnetic resonance (MR) contrast agents for imaging specific molecular targets [244]. Future approaches using peptides as components of nanoparticles might make it possible to design sensors to detect macromolecules present in specific intracellular compartments [136]. One very important implementation of nanoscale technology for clinical evaluation comes in the form of perfluorocarbon nanoparticles for molecular imaging. Several groups have demonstrated that polymeric nanospheres are capable of selectively targeting different tissues for the purpose of imaging, which also has enormous implications in targeted therapy [245]. 

## CONCLUSION

Applying nanotechnology in the pharmaceutical field has revolutionized the possibilities for drug delivery, increasing the potential for the emerging new treatments with a higher specificity and lower side effects. Recently, combining nanomaterials with different drug delivery strategies in neuroscience provide reasonable hope that the formidable barriers shielding the CNS may ultimately be overcome. Nonetheless, the wide variety of strategies reflects the inherent difficulty in therapeutic and imaging agent transport across the BBB. In fact, the effective combination of several approaches, such as encapsulation of drugs into nanoparticles conjugated with vector moieties or using micelles of Pluronic block copolymers along with Pluronic ‘‘unimers’’ for drug efflux transporter inhibition in brain capillaries, may yield promising outcomes. The abilities to package a variety of drugs in cells to affect neuro-regeneration, anti-inflammatory activities, or prevent microbial infections within the CNS have received significant attention. Nanomedicines have permitted to increase the biological and pharmacological performance of a number of drugs as well as to offer interesting alternatives for the formulation of molecules and compounds originated from the biotechnology. There is also a great potential for the development of clinical CNS nanotechnologies to participate in the treatment of neurological disorders, including multidimensional intractable disorders that have no current satisfactory treatment options. This potential stems directly from the design and control of nanotechnologies at nanoscale spatial levels, since this is the fundamental and functional building block level of the cell.

Although nanotechnology has advanced rapidly and nanoparticles are entities that can revolutionize therapy, imaging and early diagnosis of various diseases, some inherent problems need to be addressed. While developing therapeutics, attention should be given to toxicity. Coated or uncoated nanoparticles have a tendency (in varying amounts) to accumulate in the liver. Therefore, a detailed mechanism for exclusion from the body needs to be properly addressed. Some important issues during formulating nanoparticles are minimizing the batch-to-batch variations, the synthetic yield and the drug-loading effectiveness that must also be boosted to warrant practical utility of the nanoparticles. In addition, the mechanism of endocytosis and degradation pathways needs to be addressed because these are still poorly understood, despite their primary importance for clinical transition. An urgent need seems to be the safety guidelines by the governments regarding the environmental effects and the potential effects on the health of people manufacturing the nanoparticles. Despite these concerns, the near limitless possibilities for treatment strategies using nanocarriers are the exciting part in neuro-nanopharmacology.

## Figures and Tables

**Fig. (1):**
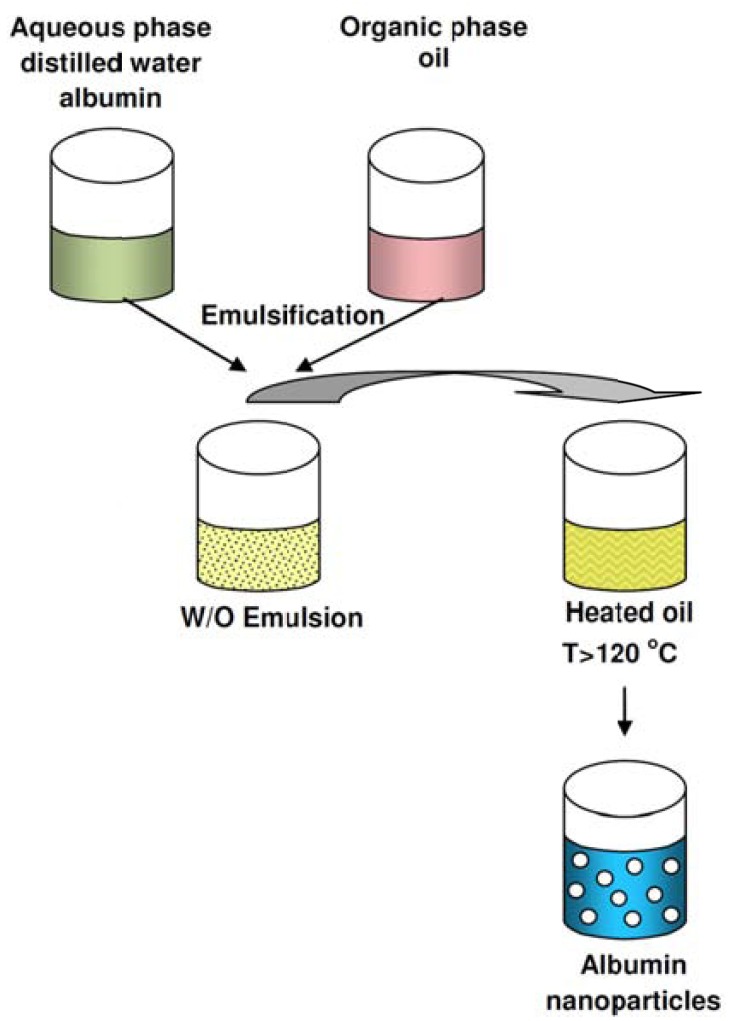
Preparation of albumin nanoparticles with emulsification method. Modified from Ref. [48].

**Fig. (2):**
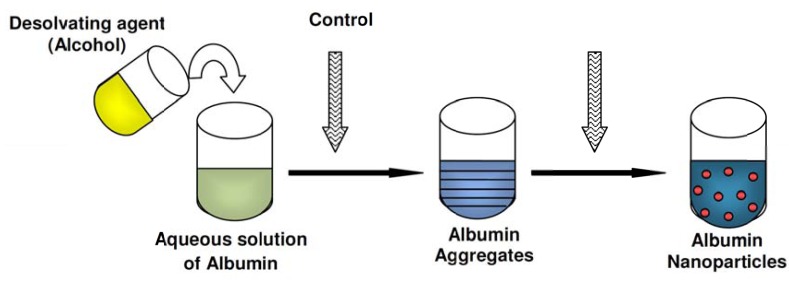
Preparation of albumin nanoparticles by coacervation method. Modified from Ref. [48].

**Fig. (3):**
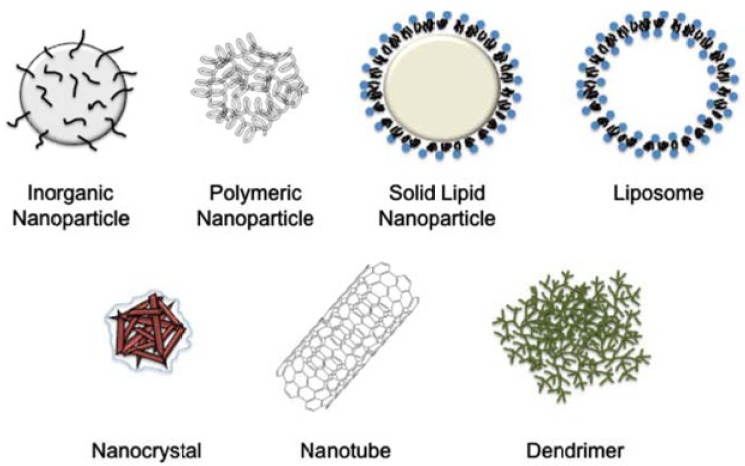
Various types of nanoparticles/ nanostructures used in biomedical research and drug delivery (adapted from [72]).

**Table 1.** Some Challenges with use of Large size Materials in Drug Delivery

Challenge	References
Poor bioavailability	[12-14]
*In vivo* stability	[15]
Solubility	[16]
Intestinal absorption	[17, 18]
Sustained and targeted delivery to site of action	[19-21]
Therapeutic effectiveness	[22, 23]
Generalized side effects	[7, 24]
Plasma fluctuations of drugs	[25, 26]

**Table 2.** Some of NPs Used for Delivery of Drugs Across the BBB (Adapted from [88])

NP Type	Drug Tested	NP size (nm)	Polymer Used/Stabilizer	Results
Solid lipid NP	Camphotericin	196.8	Soybean oil	Increased brain AUC-10.4 fold
Polysaccharide core	Albumin	60	Maltodextrin	27-fold increase in transport across *in-vitro* BBB model
Solid NP	Dalagrin	260	Polybutylcyanoacrylate	Analgesia study: increased latency by _50%
Solid NP	Valproic acid	Not evaluated	Butylcyanoacrylate/dextran 70 kDa, polysorbate-85	No increase in brain Concentrations Dalagrin,
Solid NP	Dalagrin, Kytorphin	Dextran: 288 Poly: 80-195	Polybutylcyanoacrylate/dextran 70 kDa, polysorbate-85	Analgesia study: increased latency by _50%
Solid NP	Amitriptyline	Dextran: 288 Poly: 80-195	Polybutylcyanoacrylate/dextran 70 kDa, polysorbate-85	Increased brain AUC >50%
Solid lipid NP	Doxorubicin	90	Stearic acid	Levels _1/4 of plasma after 4 hr vs. zero in brain without NP carrier
Solid NP	Dalagrin	230	Polybutylcyanoacrylate/dextran 70 kDa	Analgesia study: increased analgesia effect by _50%
Solid NP	Tubocurarine	230	Butylcyanoacrylate/dextran 70 kDa zero without carrier	Epileptiform spikes on EEG
Solid NP	Doxorubincin	270	Butylcyanoacrylate/dextran 70 kDa zero without carrier	~ 6 mg/g (brain) at 2-5 hr, vs. zero without carrier

**Table 3.** Some of Nanoparticle-based Nervous System Related Drugs for Research and Preclinical Trials (Adapted from [79])

Category	Drug Name	Indication	References
Polymeric micelles			
Biotinylated antibody-conjugated polymeric micelles	Daunomycin	Brain targeting	[175]
Polymersomes	Hemoglobin	Oxygen carrier	[176]
Dendrimers			
Polypropyleneimine dendrimers	Efavirenz	HIV infection	[177]
Poly(glycerol-succinic acid) dendrimers	Camptothecin	Various cancers	[178]
Albumin-based nanoparticles			
Cationic albumin-PEG nanoparticles	NC-1900 vasopressin fragment analog	Scopolamineinduced Memory deficits	[179]
Albumin-bound nanoparticles	Doxorubicin, methotrexate	Various cancers	[180]
Polysaccharide-based nanoparticles			
Glycol chitosan nanoparticles	Doxorubicin	Solid tumors	[181]
Metallic nanoparticles			
Aminosilane-coated iron oxide nanoparticles	Thermotherapy	Brain tumors	[182]
Starch-coated iron oxide nanoparticles	Magnetically guided mitoxantrone	Tumor Angiogenesis	[183]
Ceramic nanoparticles			
Silica-based nanoparticles	Photodynamic therapy	Various cancers	[184]
Silica crosslinked block copolymer micelles	Imaging agents, chemotherapies	Imaging, chemotherapy	[185]

HIV, human immunodeficiency virus; PAMAM, polyamidoamine; PEG, polyethylene glycol;

**Table 4.** Some of Clinically Approved Nanoparticle-based Therapeutics for Nervous System Related Diseases (Adapted from [79])

Category	Trade Name	Indication	Administration
Polymeric Platforms			
PEG-adenosine deaminase	Adagen	Severe combined immunodeficiency disease associated with ADA deficiency	i.m.
PEG-anti-VEGF aptamer	Macugen	Age-related macular degeneration	i.r.
PEG-GCSF	Neulasta	Neutropenia associated with cancer chemotherapy	s.c.
L-Glutamic acid, L-alanine, L-lysine, and L-tyrosine copolymer	Copaxone	Multiple sclerosis	s.c.
Liposomal platforms			
Liposomal verteporfin	Visudyne	Age-related macular degeneration, pathologic myopia, ocular histoplasmosis	i.v.
Liposomal cytarabine	DepoCyt	Malignant lymphomatous meningitis	i.t.
Liposomal daunorubicin	DaunoXome	HIV-related Kaposi’s sarcoma	i.v.
Liposomal morphine	DepoDur	Postsurgical analgesia	Epidural
Other platforms			
Nanocrystalline sirolimus	Rapamune	Immunosuppressant	Oral
Nanocrystalline fenofibrate	Tricor	Anti-hyperlipidemic	Oral

ADA, adenosine deaminase; GCSF, granulocyte colony-stimulating factor; HIV, human immunodeficiency virus; i.m., intramuscular; i.r., intravitreous; i.t., intrathecal; i.v., intravenous; PEG, polyethyleneglycol; s.c., subcutaneous; VEGF, vascular endothelial growth factor.

**Table 5.** Some of Nanoparticle-based Therapeutics for Nervous System in Clinical Trials (Adapted from [79])

Category	Trade Name	Indication	Administration	Status
Liposomal platforms				
Liposomal fentanyl	AeroLEF	Postoperative analgesic	Aerosol	Phase II
Liposomal vincristine	Onco TCS	Non-Hodgkin’s lymphoma	i.v.	Phase II/III
Liposomal doxorubicin	Sarcodoxome	Soft tissue sarcoma	i.v.	Phase I/II
Polymeric Platforms				
PEG-camptothecin	Prothecan	Various cancers	i.v.	Phase I/II
Other platforms				
Poly-L-lysine dendrimers	VivaGel	Antimicrobial protection from genital herpes and HIV infection	Topical	Phase I
Nanocrystalline 2-methoxyestradiol	Panzem NCD	Various cancers	Oral	Phase II
